# Ion current and action potential alterations in peripheral neurons subject to uniaxial strain

**DOI:** 10.1002/jnr.24408

**Published:** 2019-03-30

**Authors:** Fabio Bianchi, Majid Malboubi, Julian H. George, Antoine Jerusalem, Mark S. Thompson, Hua Ye

**Affiliations:** ^1^ Department of Engineering Science Institute of Biomedical Engineering, University of Oxford Oxford United Kingdom; ^2^ Department of Engineering Science University of Oxford Oxford United Kingdom; ^3^ Present address: Department of Mechanical Engineering The University of Birmingham Birmingham United Kingdom

**Keywords:** action potential, Electrophysiology, ion current, peripheral nerve, whole‐cell patch clamping

## Abstract

Peripheral nerves, subject to continuous elongation and compression during everyday movement, contain neuron fibers vital for movement and sensation. At supraphysiological strains resulting from trauma, chronic conditions, aberrant limb positioning, or surgery, conduction blocks occur which may result in chronic or temporary loss of function. Previous in vitro stretch models, mainly focused on traumatic brain injury modelling, have demonstrated altered electrophysiological behavior during localized deformation applied by pipette suction. Our aim was to evaluate the changes in voltage‐activated ion channel function during uniaxial straining of neurons applied by whole‐cell deformation, more physiologically relevant model of peripheral nerve trauma. Here, we quantified experimentally the changes in inwards and outwards ion currents and action potential (AP) firing in dorsal root ganglion‐derived neurons subject to uniaxial strains, using a custom‐built device allowing simultaneous cell deformation and patch clamp recording. Peak inwards sodium currents and rectifying potassium current magnitudes were found to decrease in cells under stretch, channel reversal potentials were found to be left‐shifted, and half‐maximum activation potentials right‐shifted. The threshold for AP firing was increased in stretched cells, although neurons retained the ability to fire induced APs. Overall, these results point to ion channels being damaged directly and immediately by uniaxial strain, affecting cell electrophysiological activity, and can help develop prevention and treatment strategies for peripheral neuropathies caused by mechanical trauma.

**Significance**

Stretch injuries to the peripheral nerve can originate from traumatic events, such as joint dislocation or limb misplacement during anesthesia. This can cause impaired motor and sensory function, leading to impaired quality of life. The mechanism linking injury to impaired nerve function is not well understood, in part due to experimental difficulties. Current in vitro models of nerve and neuron injury do not replicate the mechanical environment that cells are subjected to. Here, we use a custom‐built stretching device to apply *whole cell* uniaxial tensile deformation and simultaneously measure whole‐cell ion currents and action potential firing characteristics of sensory neurons.

## 1 INTRODUCTION

Supraphysiological stretch of 5%–20% in peripheral nerves as a result of trauma, aberrant limb positioning, or surgery, is known to cause conduction blocks (Rickett, Connell, Bastijanic, Hegde, & Shi, 2011), however, the mechanisms are not fully understood. We have previously shown how strain is transferred from tissue to cells in peripheral nerve (Bianchi, Sedgwick, Ye, & Thompson, 2018). In this work, we focus on the lower scale, investigating how whole‐cell strain affects membrane‐embedded ion channels and their ability to fire action potentials (APs).

Strain induces significant magnitude‐dependent cell depolarization (Tavalin, Ellis, & Satin, 1995), coupled with increased intracellular calcium (Gurkoff, Shahlaie, & Lyeth, 2012; Weber, Rzigalinski, Willoughby, Moore, & Ellis, 1999), which is thought to initiate mitochondrial failure (Ahmed, Rzigalinski, Willoughby, Sitterding, & Ellis, 2000) and protease activation (Iwata et al., 2004; Pettus, Christman, Giebel, & Povlishock, 1994), leading to impaired cell functionality and death. Calcium influx following injury has also shown to be tetrodotoxin (TTX)‐sensitive, indicating potential damage to sodium ion channels (Wolf, Stys, Lusardi, Meaney, & Smith, 2001). Additionally, even repeated mild trauma (strains <5%) has been shown to cause a TTX‐sensitive increase in intracellular calcium levels (Yuen, Browne, Iwata, & Smith, 2009), further indicating a direct effect of cell stretch on ion channel dynamics.

The forces regulating ion channel gating originate from the surrounding lipid membrane, suggesting a close relationship between membrane mechanics and ion channel function (Lundbæk, Birn, Girshman, Hansen, & Andersen, 1996). For example, membrane stiffening by stomatin‐like protein‐3 has been shown to finely control mechanically gated ion channel activity in sensory neurons (Qi et al., 2015). This is not limited to mechanosensitive channels, as voltage‐ and ligand‐gated channels have also been shown to be variably mechanosensitive (Phillips, Ursell, Wiggins, & Sens, 2009; Tyler, 2012).

The effect of stretch on ion channel populations has been principally investigated using localized membrane suction as a method for cell membrane deformation (Beyder et al., 2010; Morris & Juranka, 2007; Morris, Prikryl, & Joós, 2015; Wang et al., 2009). Although this allows simultaneous deformation and ion current recording, it does not closely replicate strains occurring in nerves in vivo, where the whole cell is subject to deformation.

Voltage‐activated sodium channels have been shown to respond to localized deformation, with a left‐shift in channel *I*–*V* relations, leading to reduced inactivation potential, sodium leakage, and a reduced rate of AP firing (Wang et al., 2009). Sodium channel‐1.5 current activation has been observed to follow faster dynamics under pipette‐suction deformation (Morris & Juranka, 2007), and a left‐shift in peak current and reversal potential have been shown (Beyder et al., 2010). Sodium channel activity, required for AP initiation, has been shown to depend on the mechanical state of the lipid bilayer, altered by amphiphilic treatment or cholesterol addition (Lundbaek et al., 2004). Voltage‐gated potassium channels, required for repolarization during AP firing, are also known to respond to mechanical stimuli, and their gating has been associated with the cell membrane’s mechanical state (Morris et al., 2015; Schmidt & MacKinnon, 2008). These results point to a close relationship between the mechanical environment of the cell membrane and ion channel function, suggesting that macroscopic membrane strains, such as those occurring in vivo during supraphysiological nerve elongation can cause alterations in ion channel activity leading to impaired nerve electrophysiology.

The aim of this study was to investigate the effects of whole‐cell uniaxial strain on single cell electrophysiology in DRG‐derived sensory neurons by whole‐cell patch clamping, and to quantify changes in ion channel properties during straining. We achieved this by applying strain macroscopically to the entire cell by stretching neurons adherent to a deformable substrate, and using whole‐cell patch clamping on deformed cells to evaluate the immediate changes in ion channel and AP activity.

## 2 MATERIALS AND METHODS

### 2.1 Cell culture

F11 cells (ECACC, UK), a hybrid of rat neuroblastoma and primary DRG neurons commonly used as model peripheral sensory neurons (Prucha et al., 2018), were chosen due to their ease of culture and well characterized electrophysiological properties including calcium (Kusano & Gainer, 1993), potassium (Fan, Shen, & Scheideler d, M. A., and Crain a, S. M., 1992) and TTX‐sensitive sodium (Gaudioso, Hao, Martin‐Eauclaire, Gabriac, & Delmas, 2012) ion channels. F11s were cultured on custom‐built deformable silicon substrates and differentiated in high glucose dulbecco’s modified eagle medium (ThermoFisher, UK), supplemented with 1% fetal bovine serum (ThermoFisher, UK), 1% penicillin/streptomycin, 0.5% insulin transferrin selenium (ThermoFisher, UK), 10 µM 3‐isobutyl‐1‐methylxanthine (IBMX, Sigma‐Aldrich, UK), 50 ng/ml nerve growth factor (Peprotech, UK), 2 µM retinoic acid (Sigma‐Aldrich, UK) and 0.5 mM bromoadenosine 3,5‐cyclic monophosphate. Cells were differentiated for 5 days prior to experiments and were confirmed to adhere to the deformable substrates by microscopy inspection.

### 2.2 Cell stretching

**Figure 1:**
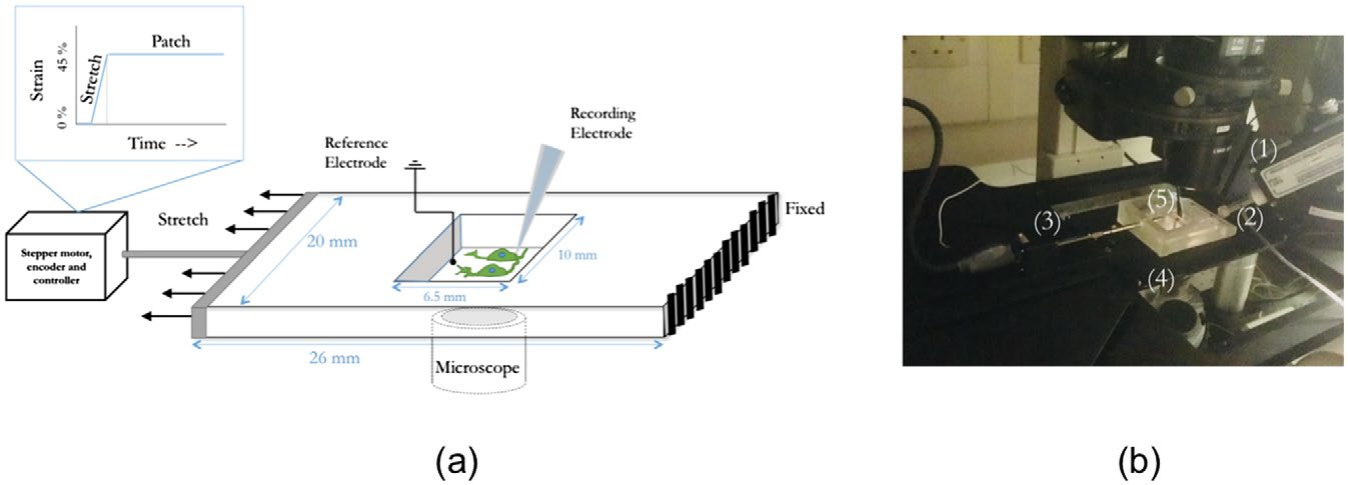
Experimental setup for cell straining (a). Neurons cultured on deformable polydimethylsiloxane substrates as subjected to uniaxial strains, and single‐cell activity is recorded simultaneously by whole‐cell patch clamping (b) Setup showing micromanipulator (1), micropipette (2), stepper motor and actuator rod (3) showing strain pulse protocol in (a), microscope stage and inverted objective (4) and deformable substrate and reference bath electrode (5) [Color figure can be viewed at wileyonlinelibrary.com]

Cells were deformed using a custom‐built substrate stretching device (Figure 1), delivering uniaxial strain to cells cultured on ultrathin (50–100 µm) polydimethylsiloxane substrates (Silex, UK), and allowing for simultaneous high‐resolution microscopy and electrophysiological recording previously used in cell‐stretching experiments aimed at discerning the response of cells to membrane stretch (Wei et al., 2018). F11 cells were seeded on deformable substrates at 5,000 cells/substrate to allow for cell proliferation during differentiation.

F11 cells were subjected to a 45% strain at 0.1 s^−1^ strain rate, and recordings were made either on cells subject to strain held at 45% (*n* = 10), or after 4 hr incubation at 37 °C and 5% CO_2_ (*n* = 7). Control recordings were taken from cells (*n* = 11) cultured on deformable membranes kept in identical conditions to stretched cells to account for substrate material effects. The stain magnitude of 45% and strain rate of 0.1 s^−1^ were chosen as a midpoint between traumatic injury modeling (faster strain rates) and chronic deformation. The strain magnitude was chosen based on previous literature, where a decrease in spontaneous neural activity was observed after approximately 40% strain (Goforth, Ren, Schwartz, & Satin, 2011). Although reports of the effect of different strain rates vary, most results show that rates as high as 30 s^−1^ (Magou, Pfister, & Berlin, 2015) and as low as 0.008 s^−1^ (Shi & Whitebone, 2006) both produce a damaging effect on neural activity. The chosen strain rate was therefore optimized for the experimental protocol and setup used.

### 2.3 Whole‐cell patch clamp recording

The experimental setup for electrophysiological recording consisted of a digidata 1550A Digitizer and a MultiClamp 700B amplifier piloted through pCLAMP 10 Software (all from Molecular Devices, CA). Glass micropipettes were pulled from thin wall borosilicate capillary tubes (BF100‐78‐10, Sutter Instruments, CA), using a Flaming/Brown micropipette puller (Model P‐1000, Sutter Instruments, CA), to a final resistance of 10 ± 1 MΩ. Pulling parameters were optimized according to previous work (Malboubi, Gu, & Jiang, 2011) in order to obtain the desired shape and surface properties of micropipettes. The intracellular solution contained: 140 mM KCl, 5 mM NaCl, 0.5 mM CaCl_2_, 2 mM MgCl_2_, 10 mM HEPES, 1 mM GTP, 2 mM ATP, with pH adjusted to 7.4 by addition of KOH and osmolarity adjusted to 300 mOsml^−1^ by glucose addition. The bath solution contained: 130 mM NaCl, 5 mM KCl, 2 mM CaCl_2_, 1 mM MgCl_2_, 10 mM glucose, 10 mM HEPES, with pH adjusted to 7.4 by addition of NaOH and osmolarity adjusted to 300 mOsml^−1^ by glucose addition. Cell capacitance was accounted for using software compensation on pCLAMP.

**Figure 2:**
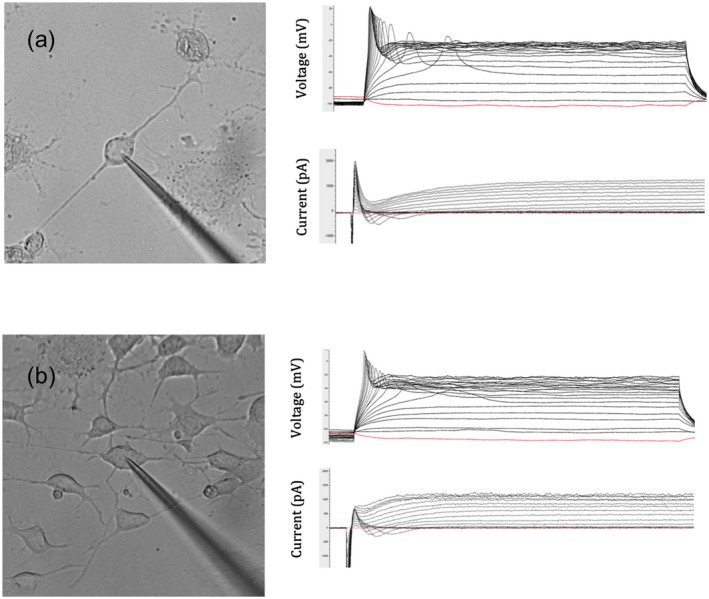
Patch clamping of F11 cells in the Unstretched (a) and Stretched (b) states, showing representative current (top) and voltage (bottom) clamp traces and images of patched cells [Color figure can be viewed at wileyonlinelibrary.com]

To evoke voltage dependent currents, cells were stimulated with a series of depolarizing pulses from −90 to + 70 mV with a step size of 10 mV and currents were recorded in voltage clamp mode (Figure 2 shows representative traces). APs were evoked in current clamp mode by a series of current pulses from −50 to +80 pA with a step size of 10 pA. Membrane resting potential (MRP) was measured upon breaking into whole cell mode with no current injection (*I* = 0). Maximum channel conductance was measured as the slope of the linear section of the *I*–*V* curve, as measured from the reversal potential point *V_r_*(*V_r_ = V*(*I* = 0)). Conductance (in nS) at each clamp voltage (*V*) was calculated as *G* = (*V* − *V*_*r*)/*I* where *I *is the ion current (in pA) at each) clamp voltage, and *V_r_*the reversal potential (in mV). Conductance was plotted against clamp voltage, and fitted with a standard Boltzmann curve, from which half‐maximal activation potential was calculated (Sontheimer & Olsen, 2007).

Cells were chosen for patch clamping based on morphology, with selected cells displaying at least three processes and distinct pyramidal neuron‐like somatas. Only cells where gigaseals were successfully formed (*±*50% of attempted cells in both unstretched and stretched conditions) were tested. Following establishment of whole‐cell patches, only cells displaying both inwards and outwards currents were used for analysis. Control cells were patched on unstretched deformable membranes, kept in the same conditions as stretched cells. Stretched cells were patched during an applied strain of 45%, immediately following stretch release, and after 4 hr recovery. Attempts were made to record ion currents and APs from the same cell before and after strain, by establishing a gigaseal in the unstrained condition and moving the micromanipulator along with the cells, so as to tentatively maintain gigaseal connection. This always resulted in gigaseal disconnection due to the mechanical disturbance from the stretch, and was therefore not possible. Comparisons of the same cell before and after stretch were therefore not possible, and all measurements were made on independent samples of cells and compared statistically.

### 2.4 Statistical analysis

Statistical analysis was carried out in PRISM v6 (Graphpad, USA). Data were tested for significance by unpaired student *t*‐test as all experimental data sets compare two separate conditions. All graphed data is reported as mean ± standard deviation. Exact *p* values are reported in each figure caption. Reported values of n represent number of cells used. For all experiments, n comes from at least three independent cultures.

## 3 RESULTS

**Figure 3:**
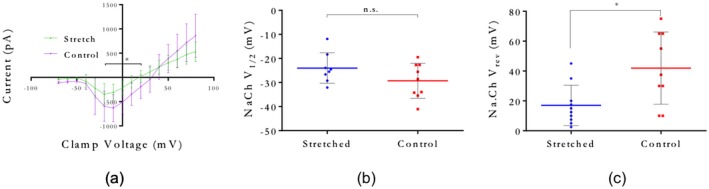
Whole‐cell patch clamp of stretched and unstretched F11 neurons, showing sodium channel dynamics. (a) Inwards sodium peak *I*–*V* plots from voltage clamp recordings. *p < 0.05 by unpaired *t* test at each clamp voltage. (b) Reversal potential for stretched and unstretched cells. *p = 0.0118. *n* = 10 (unstretched) and 9 (stretched). (c) Half‐maximum activation potential for stretched and unstretched cells. n.s. *p* = 0.1327. *n* = 8 (unstretched) and 9 (stretched) [Color figure can be viewed at wileyonlinelibrary.com]

**Figure 4:**
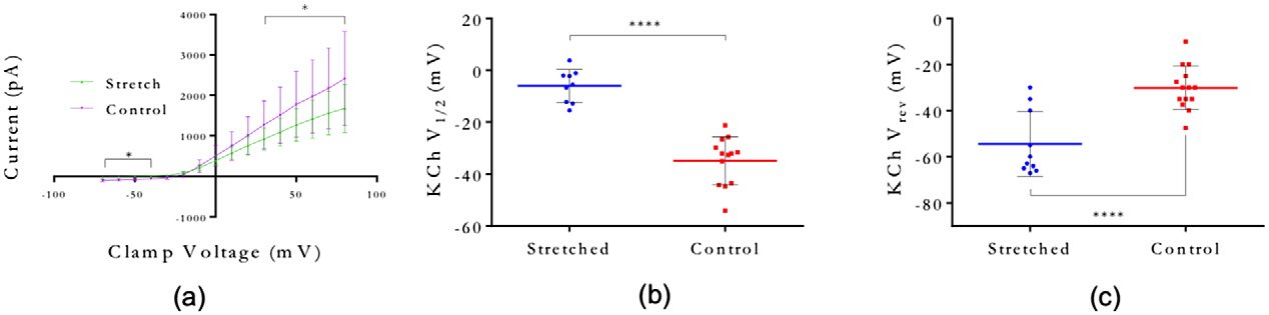
Whole‐cell patch clamp of stretched and unstretched F11 neurons, showing potassium channel dynamics. (a) Inwards potassium peak *I*–*V* plots from voltage clamp recordings. *p < 0.05 by unpaired *t* test at each clamp voltage. (b) Reversal potential for stretched and unstretched cells. ****p < 0.0001. *n* = 8 (unstretched) and 9 (stretched) (c) Half‐maximum activation potential for stretched and unstretched cells. ****p < 0.0001. *n* = 11 (unstretched) and 14 (stretched) [Color figure can be viewed at wileyonlinelibrary.com]

To evaluate alterations in ion currents and AP firing, voltage and current clamp measurements were acquired from F11 cells subjected to 45% substrate strain. Peak inwards sodium currents and outwards rectifying potassium currents were plotted against clamp voltage to produce peak *I*–*V* relations (Figures 3a and 4a). A decrease in sodium current magnitude and a left‐shift in reversal potential were found in stretched cells (Figure 3a,b). Average maximum sodium channel conductance (slope of linear section of curves in Figure 3a) was higher in control compared to stretched cells, and half maximum activation voltage was found not to be significantly changed (Figure 3c). Potassium current magnitude was also decreased during stretch, with a left‐shift in potassium reversal potential, and a lower maximum conductance compared to unstretched cells (Figure 4a,b). Potassium channel half‐maximum activation voltage was significantly right‐shifted in stretched cells compared to control (Figure 4c).

**Figure 5:**
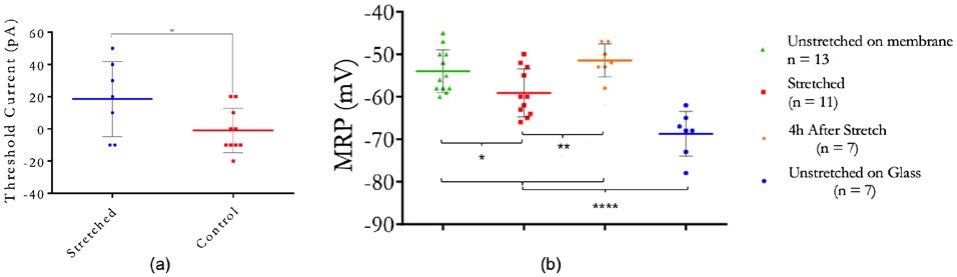
(a) Comparison of threshold current for induced AP firing. *p = 0.0455 by unpaired *t* test. *n* = 7 for stretch, *n* = 10 for control. (c) Membrane resting potentials (MRP) of cells subject to 45% substrate strain. MRPs were measured upon breaking into whole‐cell clamp, for control (unstretched) conditions, for cells during deformation, and after 4 hr incubation following deformation. MRP of unstretched (control) cells on glass to show substrate material effect. *p = 0.0329. **p = 0.0064. ****p < 0.0001. *n* = 12 (unstretched on membrane), 11 (stretched), 7 (4h after stretch), and 7 (unstretched on glass) [Color figure can be viewed at wileyonlinelibrary.com]

MRPs, measured immediately after breaking into whole‐cell configuration and in the absence of currents (*I *= 0), were not found to differ significantly between control and stretched cells, but were significantly depolarized in stretched cells 4 hr after stretch (Figure 5b).To control for substrate effects, MRP was compared between cells cultured on deformable silicone membranes and on glass, showing more negative MRP on glass. Similar changes in MRP on different substrate stiffness have been reported before (Zhang et al., 2014).

Current clamp was used to induce AP firing in F11 neurons. Stretched and unstretched cells displaying both inwards and outwards currents were induced to fire APs by incrementally increasing the clamp current magnitude. Average AP threshold current (the current clamp level needed to initiate AP firing) was significantly smaller in unstretched cells compared to stretched cells (Figure 5).

## 4 DISCUSSION

The aim of this study was to investigate the effects of whole‐cell uniaxial strains on single cell electrophysiology in sensory neurons. Ion channels, which regulate inwards and outwards flux of ions in neurons, are embedded in the cell membrane that is known to undergo high strains during cell deformation. Ion channel damage has computationally and experimentally been linked to neuronal strain (Kwong et al., 2018). Changes in the membrane mechanical environment may in turn alter the function of ion channels, leading to aberrant activity. The aim of this study was to measure changes in inwards and outwards currents and AP firing from F11 cells subject to uniaxial strain.

To evaluate changes in ion channel function during stretch, current and voltage responses of F11 DRG‐like neurons were recorded in whole‐cell patch clamp. To the best of our knowledge, the experiments reported here are the first patch clamping measurements of neurons *during *whole‐cell strain applied by substrate deformation. Previous work on the effect of mechanical deformation on ion channels used pipette suction to apply stretch to a membrane patch (Beyder et al., 2010; Morris & Juranka, 2007; Morris et al., 2015; Wang et al., 2009). This methods applies an uneven deformation, creating stress concentrations at the contact between pipette walls and cell, and providing imprecise control over stretch parameters. Furthermore, recent work (unpublished, in preparation) indicates that the observed “left‐shift” in ion current dynamics is mainly due to geometric effects at the pipette‐membrane interface and not related to ion channel damage (Kwong & Jrusalem, [Link]n preperation). By recording whole‐cell patch clamping *during *whole‐cell stretch we apply a repeatable, fully characterized strain field to a population of cells, which we believe is a closer representation of in vivo stretch conditions.

MRP was not significantly different during stretch compared to unstretched controls, although a hyperpolarization trend was observed (Figure 5b). Four hours post‐stretch, MRP was significantly depolarized compared to during stretch. Delayed depolarization has been shown to be a secondary effect of stretch (Tavalin et al., 1995), caused by calcium influx (Geddes‐Klein, Schiffman, & Meaney, 2006). We show that *during *stretch, neurons are hyperpolarized, becoming depolarized only after stretch release and incubation. A similar transient hyperpolarization followed by delayed calcium‐mediated depolarization has been reported in hypoxic neurons, where hyperpolarization by opening of potassium channels is proposed to prevent excitotoxicity (Huang, Li, Li, & Zou, 2006). Stretch may therefore similarly cause alterations in ion channel activity causing immediate protective hyperpolarization, further supported by the increased current threshold for AP initiation in strained cells (Figure 5a).

Stretched cells retained the ability to fire induced APs under current clamp, and peak parameters were not significantly altered, although the current required to initialize firing was significantly larger in strained cells in comparison to control cells (Figure 5a). This indicates neural hypoexcitability similar to that observed in anesthetized nerves, where a decrease in postsynaptic excitability suppresses neural activity (Tabatabai & Booth, 1990). If stretch similarly subdues individual neuron excitability, this would in turn affect the activity of the underlying network as a whole, potentially leading to conduction blocks and impaired sensory function, explaining the decrease in compound AP observed in strained nerves (Rickett et al., 2011).

Inwards and outwards currents are both required for AP firing. We recorded ion currents from cells subject to 45% strain, and compared them with unstrained control cells. Peak magnitudes for both sodium and potassium currents were smaller in stretched cells, compared to control (Figures 3a and 4a). Current reversal potentials, defined as the clamp voltage where ion current equals zero, were significantly hyperpolarized in stretched cells (Figures 3b and 4b). Half‐maximal activation potential (*V*_1_ *_/_*_2_) was less negative in both sodium and potassium channels (Figures 3c and 4c). A depolarized *V*_1_ *_/_*_2_ value is consistent with the measured increase in AP initiation threshold current, as a larger change in membrane potential would be needed in strained cells to achieve the same conductance (i.e., number of open channels) as in control cells.

Strain may alter the properties of the lipid membrane, in turn altering the function of ion channels. Treatment of membranes with nonionic surfactants has been shown to inhibit current flow in sodium channels (Lundbæk et al., 1996), bilayer fluidization has been linked to altered voltage‐gated ion channel function (Morris, Juranka, & Joós, 2012), and anesthetics, such as Ketamine have been shown to modulate ion channel activity by altering the lateral pressure profile of the lipid membrane (Jerabek, Pabst, Rappolt, & Stockner, 2010). Excessive strain may alter ion currents by affecting the mechanical environment where voltage‐sensing mechanisms in ion channels interact with the surrounding membrane, permanently or reversibly inhibiting the gating charge movement that regulates channel opening and closing, altering the energetic requirements of a transition between open and closed states (Sigworth, 1994). The movement of gating charges has also been shown to induce a deformation in the surrounding bilayer, demonstrating the mechanical coupling with the membrane (Krepkiy et al., 2009). Therefore, changes in conductance, half‐maximum activation voltages and altered peak *I*–*V* relations can be explained if changes in membrane mechanical properties due to stretch make the transition between closed and open less energetically viable or directly damage the gating mechanisms.

Overall, our results show that neurons undergo immediate hyperpolarization during stretch and concurrent ion channel function impairment, resulting in lower peak ion currents and depolarized activation curves and leading to higher threshold current for AP initiation. These results can help understand the onset of conduction blocks, and can be used to inform computational models of the coupling between electrophysiological function and nerve deformation, impacting preventive and treatment strategies for mechanical trauma. Voltage‐gated ion channels have been recently proposed as novel targets for neuropathic pain therapy (Tibbs, Posson, & Goldstein, 2016), and a better understanding of their association with the neural membrane mechanics can aid the development of therapies.

This study presents some limitations that can be addressed in future experiments.

The mechanical parameters tested were not an extensive representation of injury. Evaluating the effect of different strain rates, as well as smaller strain increments, would provide more information about injury mechanisms.

The electrophysiological data presented aimed at analyzing the average behavior of inwards and outwards voltage‐gated ion currents from neural membranes, capturing the whole‐cell electrophysiological behavior and more closely resembling in vivo conditions of nerve stretch. This approach did not allow for the analysis of the effect of stretch on individual families of ion currents. Further analysis of specific ion channel behavior, by selective blocking of ion channel families and by further patch‐clamping protocols (such as tail current analysis for deactivation analysis) could provide more information on the effect of stretch on specific channel types. Furthermore, using different neuronal sub‐types (for example nocioceptive vs. non‐nocioceptive sensory neurons) would allow the investigation of differential effects due to phenotype. Finally, we speculatively compare the effect of stretch to those of anesthesia and hypoxia. Testing these similarity experimentally will be a future extension of this work.

## CONFLICT OF INTEREST

The authors declare no conflicts of interest.

## AUTHOR CONTRIBUTIONS

*Methodology*, F.B., M.M., and J.H.G.; *Formal Analysis*, F.B.; *Investigation*, F.B. and M.M.; *Writing – Original Draft*, F.B. and J.H.G.; *Writing – Review & Editing*, A.J., M.S.T., and H.Y.; *Supervision*, A.J., M.S.T., and H.Y.
